# Invasive fungal infections in high-risk patients: report from TIMM-8 2017

**DOI:** 10.4155/fsoa-2018-0019

**Published:** 2018-06-14

**Authors:** Livio Pagano, Susan Mayor

**Affiliations:** 1 Institute of Hematology, Fondazione Policlinico Universitario A. Gemelli- Università Cattolica del Sacro Cuore, Rome, Italy; 2 Medical writer, London, UK

**Keywords:** antifungal resistance, azole drug monitoring, invasive fungal infections, prophylaxis

## Abstract

Trends in Medical Mycology (TIMM) is the biennial meeting of the Infectious Disease Group of the European Organisation for Research and Treatment of Cancer (EORTC) and the European Confederation of Medical Mycology (ECMM). It brings together clinicians and researchers from across the world to share the latest R&Ds in medical mycology. Despite advances in treatment, invasive fungal infections remain a major cause of morbidity and mortality in certain high-risk groups of patients, particularly in immunocompromised patients, including those undergoing solid organ transplantation and those with acute leukemia. The challenges for clinicians are now compounded by the rapid development of multidrug resistance. The latest data and approaches to identifying patients at high risk for invasive fungal infections, ensuring early diagnosis and achieving effective treatment, including when and how to use therapeutic drug monitoring with azoles, were shared with >1000 clinicians and researchers from around the world attending the eighth TIMM, held in Belgrade, Serbia, in October 2017 (TIMM-8 2017).

Patients with hematological malignancy, solid organ transplant recipients, and patients in intensive care units (ICUs) remain at particularly high risk for invasive fungal infections (IFIs) according to latest incidence data, warned Monica Slavin (Royal Melbourne Hospital, Australia) in a symposium exploring how to improve the prediction and management of patients at particular risk. She suggested that it is essential to improve awareness of patients at risk for IFIs to achieve earlier diagnosis and treatment in order to reduce the associated high mortality and risk of complications.

Considering patients with hematological malignancy, recent real-world data showed the incidence of proven or probable IFI was 11% in patients with acute myeloid leukemia (AML) [1]. Patients with acute lymphoblastic leukemia have recently been identified as a further high-risk group, with an IFI incidence of 10%, while the incidence is a somewhat lower 2–8% in chronic lymphocytic leukemia, which may reflect changes in practice including the introduction of new drugs. Transplant patients are also at risk, with an incidence of proven or probable IFI of 5–15% in patients who have undergone allogeneic hematopoietic stem cell transplantation (allo-HSCT) [1–15].

Further high-risk groups for IFI are patients admitted to ICUs and those receiving solid organ transplants. Studies show the rate of *Aspergillus* in ICU patients is 0.3–5.8%, with an overall mortality rate of over 80% [16] and during the H1N1 outbreak one study reported that 23% of ICU patients with respiratory failure developed aspergillosis [17]. The 1-year cumulative incidence of IFI in patients undergoing solid organ transplantation is highest in small bowel recipients (11.6%), followed by lung (8.6%), liver (4.7%), heart (4.0%), pancreas (3.4%) and kidney (1.3%) [18].

Slavin cautioned that the epidemiology of IFI depends on the local approach to diagnosing IFI, as well as local incidence patterns. A recent UK study in 203 patients with hematologic malignancies showed that twice weekly galactomannan (GM) surveillance gave an invasive fungal disease (IFD) incidence of 11%, which increased to 16% once targeted β-D-glucan (BDG) was used for surveillance in the same group of patients [19]. The incidence increased further to 20% with GM plus β-D-glucan testing and to 21% when all of these tests were used, including biopsy and bronchoscopy. By doing more systematic testing, we see an increase in prevalence of IFI, she noted.

Local epidemiology, including incidence of particular fungal pathogens, is another important factor. Slavin noted that *Aspergillus fumigatus* complex causes most invasive mould infections, but the predominant species varies in different locations, with *Aspergillus flavus* being more frequent in tropical climates and *Aspergillus terreus* more common in other regions, including Austria [20]. Mucormycosis ranks second in some centers, particularly in patients with sinusitis, diabetes or those on corticosteroids, while in other areas *Fusarium* spp. ranks second to *Aspergillus*. In addition, the prevalence of IFI varies between centers, which is likely to be related to activity, with higher rates in centers carrying out more transplants. The rate of antifungal resistance, including echinocandin resistance in *Candida*, and azole resistance in *Aspergillus*, also differs between centers.

## Timing of IFI risk

In terms of identifying the highest risk period, the time of first induction therapy is associated with the highest risk of IFI in patients with AML or myelodysplastic syndromes (MDS), affecting 61% of cases of AML and 36% at relapse or when resistance occurs compared with only 3% of patients at the time of complete remission [21]. Further data have shown that rates of IFI were twice as high in patients with a mean duration of neutropenia of ≥10 days (69%) compared with those with neutropenia for <10 days (31%) and much higher in patients with severe neutropenia (95%) compared with those with moderate neutropenia (5%).

The risk of IFI in patients undergoing HSCT is dynamic over time, Slavin reported. Pre-engraftment factors associated with IFI include: expected prolonged neutropenia, pre-existing prolonged neutropenia, hematological disease not in complete remission, previous HSCT, and prior IFI. Postengraftment risk factors include: grade 3–4 graft versus host disease (GVHD), chronic extensive GVHD, and viral infection [7,22–24]. Tracing the incidence of IFI over time, a large study showed that the risk of early IFI was related to whether or not a patient with acute leukemia was in complete remission whereas later IFD was associated with GVHD [7].

### Impact of new therapies on IFI risk

A further factor associated with IFI is the introduction of new therapies to treat hematological malignancies. A recent review of the use of the anti-B-cell agent ibrutinib in chronic lymphocytic leukemia and lymphoma reported a very high rate of aspergillosis, cryptococcosis, and unusual and aggressive fungal infections [25]. Patients were also receiving steroids and chemotherapy, which may have had an impact, but further case reports have found cryptococcosis and filamentous fungal infection early after starting ibrutinib. This is something that needs to be monitored and looked at in more detail. Slavin also said the time of first induction therapy is associated with the highest risk of IFI.

Checkpoint inhibitors, including ipilimumab, nivolumab, and pembrolizumab, which enhance the immune response to cancer, are starting to be used to treat hematologic malignancies. Data so far has shown no increase in IFI but these agents increase immune-related events, which are frequently managed with corticosteroids and the anti-TNF agent infliximab. This combination has been found to increase risk of IFI, with a recent case report of a patient with metastatic melanoma treated with ipilimumab plus corticosteroids and infliximab developing invasive aspergillosis (IA) [26]. Slavin suggested this was probably related to immunosuppression.

The mortality associated with IFI has been decreasing in recent years with early detection but remains quite significant. A recent French study in HSCT centers showed a 12-week mortality of 18% in patients with probably/proven aspergillosis [27], while an Italian study in patients with AML and aspergillosis the mortality was 27% [21] and 1-year mortality was approximately 70% in US patients undergoing HSCT and developing IFIs [8]. Mortality with IFI in these patients is high, and there is clearly work to be done in improving outcomes by achieving earlier diagnosis and better management, concluded Slavin.

### Optimizing antifungal prophylaxis in acute leukemia

Antifungal prophylaxis plays an important role in the management of high-risk patients with AML, Livio Pagano (Catholic University of Sacred Heart, Rome, Italy) told the meeting.

The first step in managing IFI in hematological malignancies is stratifying risk. Patients at high risk include:

- patients with AML undergoing induction chemotherapy with any of the following risk factors: neutropenia at baseline, low probability of complete remission, age over 65 years, significant pulmonary dysfunction and those with high treatment-related mortality score;
- patients with AML who have had prior IA;
- patients with AML undergoing salvage regimens for relapsed/refractory disease [28].

The risk of IFI is lower in younger patients (<45 years) with AML, those undergoing first remission-induction or consolidation chemotherapy, and patients without any risk factors for IFI.

Epidemiological data have confirmed the high risk of IFI in patients with AML, with an analysis of several studies showing a median incidence of proven or probable IFI of 8% (2–17%) and proven/probably/possible incidence of 25% (4–48%). However, a recent multicenter study in Italy suggested the incidence and mortality of candidemia in patients with hematological malignancies had fallen over the last 10 years. The incidence decreased from 1.8% in historical cohorts (1999–2003) to 0.8% in survey data from 2011 to 2015 (p < 0.001), and the attributable mortality fell from 0.6 to 0.18% (p < 0.001), with the case fatality rate decreasing from 34 to 22% (p < 0.03) [29]. This has occurred since the introduction of new antifungal agents, suggested Pagano. He noted that the reduction in incidence was particularly marked in patients with AML, where the incidence of IFI fell from 4.1 to 1.5% (p < 0.001) and the attributable mortality from 1.5 to 0.3% (p < 0.001). Greater use of prophylaxis has reduced the risk of *Candida* infection, he said.

However, IA remains a major challenge, with the risk changing across the different phases of AML treatment. A study of 391 AML patients conducted in 2001 showed that 69% of cases of IA occurred during the phase of first induction therapy [30] while a more recent study found an IFI rate of 61% during this phase [21]. This is important, argued Pagano, because the first phase of treatment can compromise the correct timing of subsequent treatment and the possibility of performing transplantation.

### The impact of antifungal prophylaxis

A recent prospective observational study of 298 patients with AML showed the impact of failing to give systemic antifungal prophylaxis during induction chemotherapy [3]. The incidence of IFI was 34.6% and the overall IFI-attributed mortality during the induction chemotherapy phase was 6.7%. In contrast, a review and meta-analysis that compared fluconazole to mold active prophylaxis to prevent IFIs in cancer patients receiving chemotherapy or HSCT showed the relative risk of proven/probable IFI was reduced by 29% (relative risk (RR) 0.71, p = 0.03), while the risk of IA was nearly halved (RR 0.53, p = 0.004) and the RR for related mortality was 0.67 (p = 0.03) [31]. Antifungal prophylaxis is mandatory in these types of patients, advised Pagano.

In terms of the choice of antifungal drugs for prophylaxis in AML, the latest guidelines from the European Conference on Infections in Leukemia (ECIL-5) update and the Infectious Diseases Society of America (IDSA) 2016 recommend:

- posaconazole – ECIL A1 recommendation; IDSA 2016 strong recommendation, high-quality evidence;
- itraconazole – B1; strong recommendation, moderate-quality evidence;
- voriconazole – BII; strong recommendation, moderate-quality evidence;
- echinocandins – CII; weak recommendation, low-quality evidence.

Fluconazole and other agents such as L-amphotericin B are not recommended [32,33]. The most positive recommendation supported by high-quality evidence is for posaconazole, said Pagano.

Is posaconazole prophylaxis during the induction phase of treatment always indicated in AML patients? The literature may seem conflicting. In fact, while a recent study using posaconazole prophylaxis during induction and salvage chemotherapy in 63 patients with AML showed no cases of proven or probable IFI occurring, one possible case, and no IFI-attributable mortality [34], a second study in 79 patients with AML given posaconazole oral suspension during induction therapy showed a rate of proven/probable/possible IFI of 30%, with one IFI-attributable death [35]. A review comparing posaconazole prophylaxis with other antifungal agents in AML patients showed markedly lower rates of proven or probable IA with posaconazole [36].

### Prophylaxis in HSCT

Moving on to transplantation, a retrospective study by the Infectious Diseases and Acute Leukemia Working Parties demonstrated a trend toward lower overall survival (hazard ratio [HR]: 1.16; p = 0.078) and higher nonrelapse mortality (HR: 1.19; p = 0.150) in recipients of allo-HSCT with preexisting IA [37]. IFIs can compromise outcomes from transplantation, Pagano cautioned. The current guidelines presented earlier all agree on fluconazole prophylaxis in the pre-engraftment phase of allo-HSCT, except IDSA 2017 which also includes a strong recommendation, based on high-quality evidence for posaconazole. In the postengraftment phase/GVHD, all guidelines give a strong recommendation for posaconazole.

Data from a single-center study in Korea following patients with AML or myelodysplastic syndrome for up to 100 days showed fungal-free survival was significantly higher in patients given posaconazole for primary antifungal prophylaxis during remission induction chemotherapy compared with those given fluconazole (p = 0.004) [38]. It is uncertain whether posaconazole prophylaxis during remission induction chemotherapy might influence the characteristics of IFIs developed during consolidation chemotherapy or HSCT, explained Pagano. He suggested that further studies are needed to investigate the changes in fungal colonization and incidence density of IFIs during the period from remission induction chemotherapy to HSCT.

A recent study showed a long-lasting protective effect of posaconazole prophylaxis in patients with AML receiving allo-HSCT. The study included 284 patients, with 40% (114) receiving posaconazole prophylaxis during first induction or salvage chemotherapy treatments, 32% (90) treated with itraconazole and 28% (80) with fluconazole. A total of 17 patients receiving itraconazole or fluconazole and five of those given posaconazole developed probably/proven IFI and were excluded from analysis. The patients then underwent allo-HSCT, all with fluconazole prophylaxis. Results showed higher rates of proven/probable IFI in those treated with itraconazole or fluconazole during induction or salvage therapy compared with those given posaconazole (14 vs 4%). Fungal-free survival was significantly higher at 1 year post-HSCT in those who had previously received posaconazole prophylaxis compared with those treated with itraconazole or fluconazole (p = 0.012) [39]. There was a marked advantage with posaconazole, commented Pagano. He suggested that this potentially may be due to the pharmacokinetics of posaconazole, with studies suggesting that it accumulates in cell membranes and remains there with a long half-life. Intracellular posaconazole can serve as an important antifungal reservoir and membrane-bound posaconazole can transfer to fungal cells resulting in growth inhibition.

Summing up, Pagano concluded that antifungal prophylaxis is essential in high-risk patients. Posaconazole prophylaxis remains the gold standard during first induction chemotherapy in AML patients and preliminary data from real-life studies seem to show that giving posaconazole prophylaxis during the induction phase appears to provide protective effects during consolidation and transplant procedures.

### IA remains important infection in solid transplant recipients

New results from the Swiss Transplant Cohort Study reported as a late-breaking abstract at congress [40] showed IA remains a rare but important infection among patients undergoing solid transplantation with higher rates in heart and lung transplants.

The study retrospectively analyzed data for all solid organ transplant recipients at six Swiss transplant centers between May 2008 and December 2014, assessing the incidence and clinical outcomes in patients developing proven or probable IA. Researchers identified patients with IA as index cases and matched these with controls (one–three ratio) based on type of solid organ transplantation, time and location of transplant procedure.

Results showed that a total of 70 of the 3035 patients (2.4%) receiving solid organ transplants developed IA. The incidence was highest in heart (7.1%) and lung (8.3%) transplants. The median time between transplantation and onset of IA was 100 days but with a wide time range (interquartile range (IQR): 15–275 days) and was shorter in lung transplants (median: 11 days, IQR: 5–103) and liver transplants (median: 18 days, IQR: 9–122).

IA was associated with high mortality, with 16 of 70 (22.9%) patients developing the infection dying by 12 weeks. Univariate analysis identified liver transplantation as a risk factor for 12-week mortality (HR: 33.7, 95% CI: 4.01–283.51), followed by bacterial co-infection (HR: 2.74; 95% CI: 1.02–7.37).

Using nested case–control data, the research group identified the major significant predictors of IA as prior bacterial infection (odds ratio [OR]: 4.6; 95% CI: 2.23–9.57; p < 0.001), respiratory viruses (OR: 11.1; 95% CI: 3.06–40.15; p < 0.001), cytomegalovirus (OR: 2.91; 95% CI: 1.3–6.54; p = 0.01) and fungal pathogens (OR: 2.6; 95% CI: 1.10–6.11; p = 0.03). In addition, intravenous administration of glucocorticosteroids and renal failure were further risk factors for IA.

IA remains a rare but important complication in solid organ transplant recipients, affecting primarily heart and lung transplants, said lead author Dionysios Neofytos (University Hospital of Geneva, Geneva, Switzerland). He added that co-infection with other pathogens – mainly bacterial and viral – appeared to increase susceptibility to IA and reduce 12-week survival.

### Potential new biomarker for IA

Looking to the future, a small study suggested that urine testing for triacetylfusarinine C (TAFC), a fungal siderophore produced by *Aspergillus fumigatus*, might offer a potential new biomarker for diagnosis of IA [41].

The Austrian study prospectively analyzed same day blood and urine samples for patients with hematological malignancies on a twice-weekly basis from 2012 to 2015. Researchers retrospectively measured TAFC, GM and urine creatinine levels in 44 samples from a total of 24 patients (eight with AML, four with acute lymphocytic leukemia, three non-Hodgkin lymphoma and nine others). Seven of the patients had probable IA, one had possible IA, and 16 had no IA. TAFC/creatinine index values were calculated by analyzing receiver operating characteristics curve and values ≥ 3 were considered positive, as were GM/creatinine index values ≥ 0.25.

Results for per patient receiver operating characteristics curve analyses showed that AUC for TAFC/creatinine for probable IA versus no IA was 0.835 (95% CI: 0.585–1.000; p = 0.012). AUC per sample for patients with probable IA versus no IA was 0.882 (95% CI: 0.771–0.933; p < 0.001).

Reporting the findings, lead author Juergen Prattes (University of Graz, Austria), said that the TAFC/creatinine sensitivity for probable versus no IA based on per patient analysis was 0.86 (95% CI: 0.49–0.97), while the specificity was 0.88 (95% CI: 0.64–0.97). Patients with a TAFC/creatinine index value more than or equal to three were nearly seven-times more likely to have probable IA than those with lower values (positive likelihood ratio for probable vs no IA: 8.86; 95% CI: 1.81–25.96).

*“Reliable biomarkers for IA in urine would offer several advantages in clinical practice, including being easily repeatable and using noninvasive sampling,”* said Prattes. He added, *“TAFC/creatinine index showed promising results in this study for diagnosis of IA.”* The sensitivity and specificity were similar to those previously reported for GM determination in bronchoalveolar lavage samples, which is the current gold standard test for diagnosing AI.

### Real-life experience with newer formulations of posaconazole

Posaconazole is recommended for prophylaxis in patients at risk for invasive fungal infections by several sets of guidelines, but many of these were developed before oral and intravenous formulations became available. Monica Slavin (Royal Melbourne Hospital, Melbourne, Australia) reviewed emerging studies and real-life evidence on how these newer formulations were performing in clinical practice.

Several studies published in the last year have reported on use of the tablet formulation of posaconazole, largely in hematological malignancies and patients undergoing HSCT. These real-life studies demonstrated that better trough levels were achieved with posaconazole tablets compared with suspensions. A German registry in which 95% of patients were treated with the posaconazole tablet and 5% with the intravenous formulation reported serum levels of 1000–1600 ng/ml [42].

Slavin noted that some studies have shown a higher proportion of patients with subtherapeutic levels than might have been expected but cautioned that this appeared to be associated with lack of systematic measurement, with clinicians being more likely to measure drug levels in those patients where there was clinical concern. One study showed 17% of patients had subtherapeutic levels of posaconazole, defined as <700 ng/ml for prophylaxis of aspergillosis [43]. A US study found subtherapeutic levels in 15% of patients receiving posaconazole tablets for prophylaxis and in 56% of those for treatment (subtherapeutic level <1000 ng/ml). In the treatment group, 15% had breakthrough IFI, with one in four of these having subtherapeutic levels [44]. She cautioned that the levels were not taken systematically and varying proportions of patients had their levels measured, adding that breakthrough IFIs were not limited to patients with subtherapeutic levels.

The intravenous formulation of posaconazole can be useful in various situations that commonly occur in patients with hematological malignancies, including patients with erratic gastrointestinal absorption, grade 3 or 4 GVHD, severe mucositis, neutropenic enterocolitis, poor tolerance to oral medications, and those who are intubated or in ICUs. A recent study with IV posaconazole as prophylaxis in AML/HSCT showed that the mean steady-state plasma drug concentration, C_avg_, was 1500 ng/ml. IFI occurred in only 1% of patients (3/237).

Reporting on the Australian experience of IV posaconazole in a named patient program for patients with hematological malignancy undergoing allo-HSCT, Slavin said that there had been no cases of breakthrough IFI in the 52 patients included. The median trough level was >1000 ng/ml, although trough level was not measured in all patients. There was no difference between starting and completing therapy in levels of ALT, total bilirubin, or creatinine clearance, but four patients discontinued due to potential concerns about abnormal liver function and one discontinued for thrombocytopenia, although none of these resolved on stopping posaconazole so this may have been unrelated [45].

Summing up, Slavin said that the real-life data provide indications on how to use the new formations of posaconazole. She noted that better trough levels were attained with the tablet than with the suspension. IV posaconazole achieved levels >1000 ng/ml in hematology patients, but she cautioned they may be lower in ICU patients. She suggested that the threshold for switching from the oral to the IV formulation should be evaluated, particularly with the availability of the tablet. She recommended monitoring levels with the new formulations when treating IFIs, in ICU patients and in patients with GVHD, post lung transplant in cystic fibrosis patients and where there are concerns about possible toxicity.

### Therapeutic drug monitoring of azoles

Therapeutic drug monitoring (TDM) of azoles is a hot topic, suggested Russell Lewis (Infectious Diseases Unit, Sant Orsola-Malpighi Hospital, Bologna, Italy) during a symposium exploring the latest evidence and new developments in the field. He noted that there had been a dramatic increase in published studies on azole TDM over the last few years. Several guidelines recommend TDM for antifungals, although they primarily focus on IA, despite there being no definitive data from clinical trials addressing its use in clinical situations and the fact that azoles were shown to be effective in clinical trials without TDM.

Triazole antifungals can display unpredictable pharmacokinetics in critically ill or severely immunocompromised patients making it difficult to predict whether patients will achieve safe and effective drug exposures. With the high morbidity and mortality associated with IFDs, many treatment guidelines have recommended TDM for patients receiving mould-active triazole therapy, even though the introduction of newer posaconazole formulations and isavuconazole has renewed debate whether routine TDM is necessary.

Ensuring an effective dose of antifungal therapy is an essential component in the therapeutic success, argued Lewis. For most drugs, the clinical effect is readily observable in the clinical or biochemical effect, but this is not the case for antifungals or antibiotics. TDM is also useful for antifungals, because specific patient groups in which they are commonly used, including those with hematological diseases and those who are critically ill, have altered pharmacokinetics. They may also have complications such as vomiting or diarrhea leading to impaired oral bioavailability and altered drug distribution and drug clearance.

The following is the rationale for TDM for different azoles:

- TDM may be useful for voriconazole, because there is substantial pharmacokinetic (PK) variability, and it has a narrow therapeutic window that is defined in humans. Lewis recommended that minimum plasma drug concentration, C_min_, monitoring should be started at day 2–5 in every patient treated with voriconazole and repeated after 7 days to confirm patients are in the target range (1–6 mg/l).
- Two new formulations of posaconazole, a gastro-resistant tablet and an intravenous formulation, have been developed to overcome the bioavailability problems with the oral suspension. Lewis reported that a study in leukemia patients switching from the suspension to tablets showed an increase in serum drug levels without clinically relevant hepatotoxicity [46]. He noted that some subsets of patients treated with posaconazole tablets have shown subtherapeutic concentrations but this was often associated with risk factors such as diarrhea or high body mass index [47].

Real-life studies have shown suboptimal posaconazole exposure with tablet or IV formulation was associated with very low rates of breakthrough IFI (0.0–6.7%) [44,48]. In addition, there is no association between increasing posaconazole exposure and liver injury or QTc prolongation [49]. Overall, Lewis considered that TDM for posaconazole could be useful because the azole shows substantial PK variability and the therapeutic window in humans is well defined, but he noted that whether or not the drug has a narrow therapeutic window is not clear.

- Isavuconazole is rapidly absorbed and has a very long half-life, with less pharmacokinetic variability compared with voriconazole. It shows substantial PK variability, and the therapeutic window is not defined in humans. In addition, it is not clear if it has a narrow therapeutic window.
- Fluconazole shows substantial variability in some populations, including critically ill patients with sepsis and obese patients, potentially leading to subtherapeutic exposure. However, the monitoring strategy is unclear and it has a broad therapeutic window.

## The importance of monitoring correctly

It is essential that azole monitoring is performed correctly, advised Isabel Spriet (University Hospitals Leuven and KU Leuven, Belgium). She noted that it is important to measure trough levels just before a patient is given their next dose of an azole and not simply when other blood samples are taken or when an antifungal agent is already being infused. Peripheral venipuncture should be used to collect a blood sample for monitoring, with one study showing that falsely elevated vancomycin plasma concentrations were obtained after sampling from a central venous implantable catheter [50].

The accuracy of the analytical method used for TDM is also important. A recent study analyzing 5-year results of an international proficiency testing program for measurement of antifungal drug concentrations showed that one in five (19.2%) analyses were inaccurate, with the laboratory performing the test being the only factor clearly associated with inaccuracies [51]. ECIL-6 guidelines recommended that laboratories participate in ongoing proficiency testing programs to identify sources of error and improve analytical methods, Spriet advised. She concluded that key outstanding questions remain to be answered with further research: will TDM affect clinical decision-making and provide more information than sound clinical judgment alone?

### Failure of antifungal therapies is multicausal

Resistance to antifungal agents is increasing but the clinical relevance is currently unclear and additional drivers contribute to clinical failure in patients developing serious fungal infections, warned Matteo Bassetti (University of Udine, Italy). Supporting this view, he cited a recent study comparing treatment with echinocandins and fluconazole in patients with candidemia from a urinary tract source. Results showed that nearly a third (29.7%) of patients were nonsusceptible to fluconazole (38/128 episodes) while only 5.5% (7/128) were resistant to echinocandins. However, there was no difference in clinical failure with the two antifungal treatments (17.1% with fluconazole vs 20% with echinocandins) [52]. Further analysis showed that the only risk factor associated with clinical failure was an early urologic procedure (adjusted odds ratio: 0.08; 95% CI: 0.02–0.31; p < 0.001).

Exploring further factors associated with clinical failure in patients with serious fungal infections, Bassetti noted that development of septic shock, failure to provide adequate source control, and inadequate antifungal therapy within the first 24 h of infection all contribute to high mortality. A study in patients with abdominal candidiasis showed that those receiving inadequate antifungal therapy and those developing septic shock where the source of their infection was not controlled had a very high hospital mortality rate of over 60% [53].

Site of infection, high fungal burden, underlying disease, source control and development of biofilm are also important factors associated with clinical failure of antifungal agents, he told the meeting. A study in 294 patients with *Candida* bloodstream infections showed much higher mortality in patients with biofilm-producing isolates (70.0%) compared with those with isolates not producing biofilm (45.7%, p < 0.001) [54].

Summing up, Bassetti concluded that *in vitro* susceptibility to antifungal agents is not the only factor in determining the outcome of patients with fungal infections. Other important factors include site of infection, high fungal burden, underlying disease, source control, development of biofilm and an antifungal drug's pharmacokinetics and pharmacodynamics.
